# Linking urban park soundscape cognitive image to recreational visitors’ perceived restorativeness: the mediating role of emotional pleasure

**DOI:** 10.3389/fpubh.2026.1891815

**Published:** 2026-07-15

**Authors:** Chenyi Liao, Junlong Yang, Jiajian Li, Ziyu Zhang, Mengyao Zheng, Zhendong Huang, Ji Li

**Affiliations:** 1 College of Management, Zhongkai University of Agriculture and Engineering, Guangzhou, Guangdong, China; 2 School of International Relations, Yonsei University, Seoul, Republic of Korea; 3 Yonsei University, Seoul, Republic of Korea; 4 Department of Rehabilitation Medicine, The Affiliated Panyu Central Hospital, Guangzhou Medical University, Guangzhou, Guangdong, China; 5 College of Bioscience and Biotechnology, Hunan Agricultural University, Changsha, Hunan, China

**Keywords:** emotional pleasure, mediating pathway, perceived restorativeness, SOR framework, soundscape cognitive image, urban park soundscape

## Abstract

**Introduction:**

Accelerating urbanization has intensified psychological stress and mental health problems among urban residents. As accessible natural spaces for leisure and recreation, urban parks have received increasing scholarly attention for the potential restorative effects of their soundscapes. Existing studies have mainly focused on the restorative benefits of visual landscapes or examined only the direct relationship between soundscapes and perceived restorativeness. However, the potential psychological pathway through which soundscape cognitive image is associated with perceived restorativeness via emotional pleasure has not been sufficiently clarified. Based on the stimulus-organism-response framework, this study constructed a model linking soundscape cognitive image, emotional pleasure, and perceived restorativeness.

**Methods:**

Twelve urban parks in Foshan, China, were selected as case study sites. A field questionnaire survey was conducted among recreational visitors, and 300 valid responses were collected. Python 3.10 was used for data processing and descriptive analyses, and AMOS 26.0 was used for structural equation modeling and mediation tests.

**Results:**

The results showed that soundscape cognitive image was positively associated with emotional pleasure. Soundscape cognitive image was positively associated with the being away, fascination, and compatibility dimensions of perceived restorativeness, whereas its direct relationship with coherence was not significant. Emotional pleasure was positively associated with being away and compatibility, was not significantly associated with fascination, and showed a significant negative association with coherence. Bootstrap analysis further indicated that emotional pleasure served as a significant indirect pathway between soundscape cognitive image and being away, compatibility, and coherence. The indirect pathway for coherence was negative and the total relationship was not significant, suggesting that this result should be interpreted cautiously. No significant indirect pathway was found for fascination.

**Discussion:**

This study extends the application of the stimulus–organism–response framework to urban park soundscape research by showing how visitors’ subjective soundscape cognition is related to perceived restorativeness through emotional pleasure. Because the study used cross-sectional self-report data, the findings should be interpreted as evidence of statistical associations and potential psychological pathways rather than as proof of causal effects. The results provide empirical references for soundscape evaluation and health-oriented urban park planning.

## 1 Introduction

Global urbanization has reshaped land-use patterns, increased population density, and intensified pressure on urban green infrastructure. Evidence from rapidly urbanizing cities such as Kuala Lumpur shows that land-use and land-cover changes and alterations in urban water patterns may weaken green and blue infrastructure and aggravate environmental risks (1, 2). These examples indicate that urban development is not only a process of spatial expansion but also a challenge for environmental governance and public health. In China, urbanization has also continued rapidly, and the urbanization rate is expected to reach 75% by 2035 (3). The rapid concentration of urban populations and the increasingly fast pace of urban life have led to a continuous rise in psychological stress among residents. Mental health problems, including depression, anxiety disorders, and insomnia, have become increasingly prevalent and now represent major public health challenges (4). Humans have an innate affinity for the natural environment, and this biological tendency makes contact with nature an effective way to relieve mental fatigue and restore cognitive functioning (5). Previous studies have shown that even brief exposure to natural environments can significantly improve attention, enhance emotional states, and reduce physiological stress responses (6). In this context, urban parks, as highly accessible green infrastructure in cities, not only perform ecological regulation functions but also serve as important places for leisure, recreation, social interaction, and physical and mental recovery (7, 8).

In recent years, the health promotion functions of urban parks have received extensive scholarly attention. Public investment in urban parks has also increased at the national level, with the expectation that they will assume broader public health service functions (9). However, existing studies have mainly focused on the restorative benefits of visual landscapes, such as the effects of green space coverage, plant configuration, and spatial layout on mental health. By contrast, the restorative role of soundscape, as a key environmental factor, has received relatively less attention (10). The concept of soundscape was first proposed by the Finnish geographer J. G. Granö in 1929 (11) and was later systematically developed by the Canadian composer Schafer. It is commonly defined as “the acoustic environment as perceived by individuals” (12, 13). As an important ecosystem service, natural soundscapes play an important role in human physical and mental health (14). Physiological studies have confirmed that natural sounds, such as birdsong and flowing water, can increase alpha-wave activity, reduce beta-wave activity, regulate heart rate and blood pressure, and thereby help relieve stress and anxiety (15, 16). Psychological studies have also found that natural sounds can induce positive emotions and enhance environmental pleasure and comfort (17, 18).

Urban parks can respond to these challenges by providing multiple ecosystem and public-health services, including ecological regulation, stormwater buffering, heat mitigation, physical activity opportunities, social interaction, and everyday psychological recovery. In addition to visual landscape features, the acoustic environment perceived by park visitors forms an important part of these services. Previous research on public parks has shown that pleasant sounds are closely related to visitors’ positive environmental experiences, and that natural and socially meaningful acoustic elements can shape how people evaluate park environments (19). Therefore, examining how visitors cognitively evaluate urban park soundscapes is important for both restorative-environment research and health-oriented park management.

Although the health value of soundscapes has been preliminarily confirmed, several limitations remain in existing research (20, 21). On the one hand, soundscape studies in China and abroad have mainly focused on soundscape evaluation, sound design, and soundscape preference, while relatively limited attention has been paid to restorative mechanisms (22, 23). On the other hand, the small number of studies concerning soundscape restorativeness have mostly adopted experimental methods. Large sample empirical tests in real natural environments are still lacking, and the internal psychological pathways through which soundscape perception is associated with perceived restorativeness have not been systematically revealed (24). In particular, emotional pleasure may serve as an important organismic response between environmental stimuli and restorative psychological responses, but its role in the relationship between soundscape cognitive image and perceived restorativeness remains insufficiently clarified (25).

Recent restorative soundscape studies have increasingly emphasized that restoration in parks is shaped by combined auditory, visual, contextual, and individual factors rather than by sound alone. Guo et al. showed that audio-visual interaction and visitor characteristics are associated with perceived soundscape restorativeness in urban parks (26). Li and Liu further demonstrated that visual and auditory environments in parks are linked to landscape preference, emotional state, and perceived restorativeness, and that such relationships may vary across park types and environmental combinations (27, 28). Beyond urban parks, Yang and Zhang found that rural soundscape perception was related to environmental restoration through affective place-related variables (29). These studies indicate that soundscape restoration should be examined as a perception-based and emotion-related process embedded in specific environmental contexts.

Perceived restorativeness refers to the process and outcome through which individuals perceive and interact with the restorative characteristics of an environment, leading to mental and cognitive recovery (30). Attention Restoration Theory, proposed by Kaplan and Kaplan, is a core theory in this field. It suggests that environments characterized by being away, fascination, coherence, and compatibility can help individuals recover from directed attention fatigue (31, 32). Existing studies have shown that visual landscapes can enhance perceived restorativeness by inducing positive emotions. However, as an important component of multisensory experience, the specific pathway through which soundscape cognitive image is associated with perceived restorativeness remains unclear (33, 34). In particular, empirical evidence remains limited regarding how urban park soundscape cognitive image is associated with the four subdimensions of perceived restorativeness through emotional pleasure.

The stimulus-organism-response model, also known as the SOR model, posits that environmental stimuli are associated with individuals’ internal psychological and physiological states, which are subsequently related to behavioral or cognitive responses. In tourism and environmental psychology, the SOR model has been widely applied to examine the relationships among landscape perception, tourist experience, and behavioral intention (35). Recent studies have shown that natural soundscapes, as important environmental stimuli, are associated with visitors’ emotional states and recreational experiences (36). However, applications of the SOR model to restorative soundscape research in urban parks remain relatively limited. In particular, few studies have systematically examined soundscape cognitive image as the stimulus, emotional pleasure as the organismic response and potential mediating pathway, and perceived restorativeness as the response outcome (37, 38).

Despite these advances, three research gaps remain. First, restorative-environment studies have paid more attention to visual landscape attributes than to visitors’ cognitive evaluation of soundscapes. Second, many soundscape studies have examined sound preference or the direct relationship between soundscape perception and restoration, whereas the affective pathway linking soundscape cognitive image to perceived restorativeness remains insufficiently clarified. Third, limited field-based evidence is available from multiple urban parks that distinguishes the four dimensions of perceived restorativeness. To address these gaps, this study has two objectives. First, it examines the associations among urban park soundscape cognitive image, recreational visitors’ emotional pleasure, and the four dimensions of perceived restorativeness, namely being away, fascination, coherence, and compatibility. Second, it tests whether emotional pleasure serves as a potential mediating pathway between soundscape cognitive image and these restorative dimensions within the SOR framework. The contribution of this study is threefold: it extends the SOR framework to restorative soundscape research, clarifies the selective role of emotional pleasure in different restorative dimensions, and provides field-based evidence from 12 urban parks for health-oriented soundscape evaluation and planning.

## 2 Theoretical framework and research hypotheses

### 2.1 SOR framework and formal emotional-state specification

The stimulus–organism–response (SOR) framework suggests that environmental stimuli are associated with individuals’ internal psychological states, which are subsequently related to cognitive or behavioral responses (35). In the context of urban parks, soundscape perception is not merely exposure to sound pressure levels; it also includes visitors’ recognition of sound sources, evaluation of acoustic comfort, and interpretation of the relationship between sounds and place meanings. Therefore, this study treats the cognitive image of urban park soundscapes as the environmental stimulus, emotional pleasure as the organismic affective state, and perceived restorativeness as the psychological response.

Emotion is often conceptualized through the dimensions of pleasure and arousal. Previous research indicates that individuals may experience both pleasure and arousal when engaging in environmental or recreational behaviors (39), and Russell’s circumplex model further explains the organization of affective states through pleasure and arousal dimensions (40). In this study, emotional pleasure refers to the comfort, enjoyment, and satisfaction experienced by visitors when perceiving urban park soundscapes. During the preliminary survey and subsequent data screening and model-fitting procedures, the emotional arousal dimension did not show sufficient compatibility with the model used in this study. Therefore, the formal research model includes emotional pleasure as the emotional-state variable. This specification clarifies the analytical scope of the present study and does not imply that arousal is unimportant in all soundscape contexts.

Gross noted that emotion reflects the relationship between individuals and external environments, and that different environments may trigger different emotional responses (41). In soundscape contexts, sound sources and the information they convey are important factors shaping soundscape perception (42). From a health perspective, the appropriate use and perception of sound can improve human experience (43), and positive soundscapes are related to mental health and quality of life (44). These studies suggest that a favorable cognitive image of urban park soundscapes may be associated with more positive emotional evaluations. Accordingly, the following hypothesis is proposed:

*H1*: The cognitive image of urban park soundscapes is positively associated with recreational visitors’ emotional pleasure.

### 2.2 Soundscape cognitive image and perceived restorativeness

Perceived restorativeness refers to visitors’ perception of environmental characteristics that support psychological recovery and includes four dimensions: being away, fascination, coherence, and compatibility (30–32). Soundscape cognitive image reflects visitors’ overall cognitive evaluation of the urban park sound environment. Recreational visitors’ perception of soundscapes is a complex cognitive process involving associations, memories, and reflections related to the surrounding environment. Satisfaction with the soundscape may therefore be related to overall environmental satisfaction (45, 46). In addition, visitors’ reflection on sound-related issues in urban parks can strengthen their environmental values and further consolidate emotions and attitudes shaped by soundscape perception (47, 48).

Recent studies on restorative soundscapes and audio-visual environments further indicate that park restoration is shaped by auditory, visual, contextual, and individual factors. Audio-visual interaction and visitor characteristics have been shown to be associated with perceived soundscape restorativeness (26), while park visual and auditory environments are related to landscape preference, emotional state, and perceived restorativeness (27, 28). Evidence from rural soundscape research also suggests that soundscape perception may be related to environmental restoration through affective place-related variables (29). Based on these findings and the original soundscape perception literature (42–44), a favorable cognitive image of urban park soundscapes may be associated with stronger restorative perceptions. Because the four dimensions of perceived restorativeness represent different aspects of restoration, the relationships should be tested separately. Accordingly, the following hypotheses are proposed:

*H2a*: The cognitive image of urban park soundscapes is positively associated with being away.

*H2b*: The cognitive image of urban park soundscapes is positively associated with fascination.

*H2c*: The cognitive image of urban park soundscapes is positively associated with coherence.

*H2d*: The cognitive image of urban park soundscapes is positively associated with compatibility.

### 2.3 Emotional pleasure and perceived restorativeness

Tourism and environmental psychology studies have shown that tourists’ emotional experiences are an important component of environmental experience and evaluation (49). Soundscapes also influence recreational visitors’ psychological stability, place experience, and behavioral choices. Previous studies have emphasized complex interactions among soundscapes, human emotions, and sustainable environments (50), and have shown that soundscape perception can shape visitors’ experience in urban and historical public spaces (51). Place attachment research further indicates that emotional bonds with places are related to place satisfaction, pro-environmental behaviors, place meaning, and social well-being (52–55). Natural sounds in urban parks may also encourage social interaction and positive recreational experience (56).

On this basis, emotional pleasure may be associated with visitors’ perceived restoration. A pleasant emotional state may help visitors feel relaxed, temporarily distant from daily routines, more attentive to environmental features, and more compatible with the park setting. However, because being away, fascination, coherence, and compatibility represent different dimensions of restorative perception, the relationship between emotional pleasure and each dimension should be empirically tested rather than assumed to be uniform. Accordingly, the following hypotheses are proposed:

*H3a*: Emotional pleasure is positively associated with being away.

*H3b*: Emotional pleasure is positively associated with fascination.

*H3c*: Emotional pleasure is positively associated with coherence.

*H3d*: Emotional pleasure is positively associated with compatibility.

### 2.4 Mediating role of emotional pleasure

**Figure 1:**
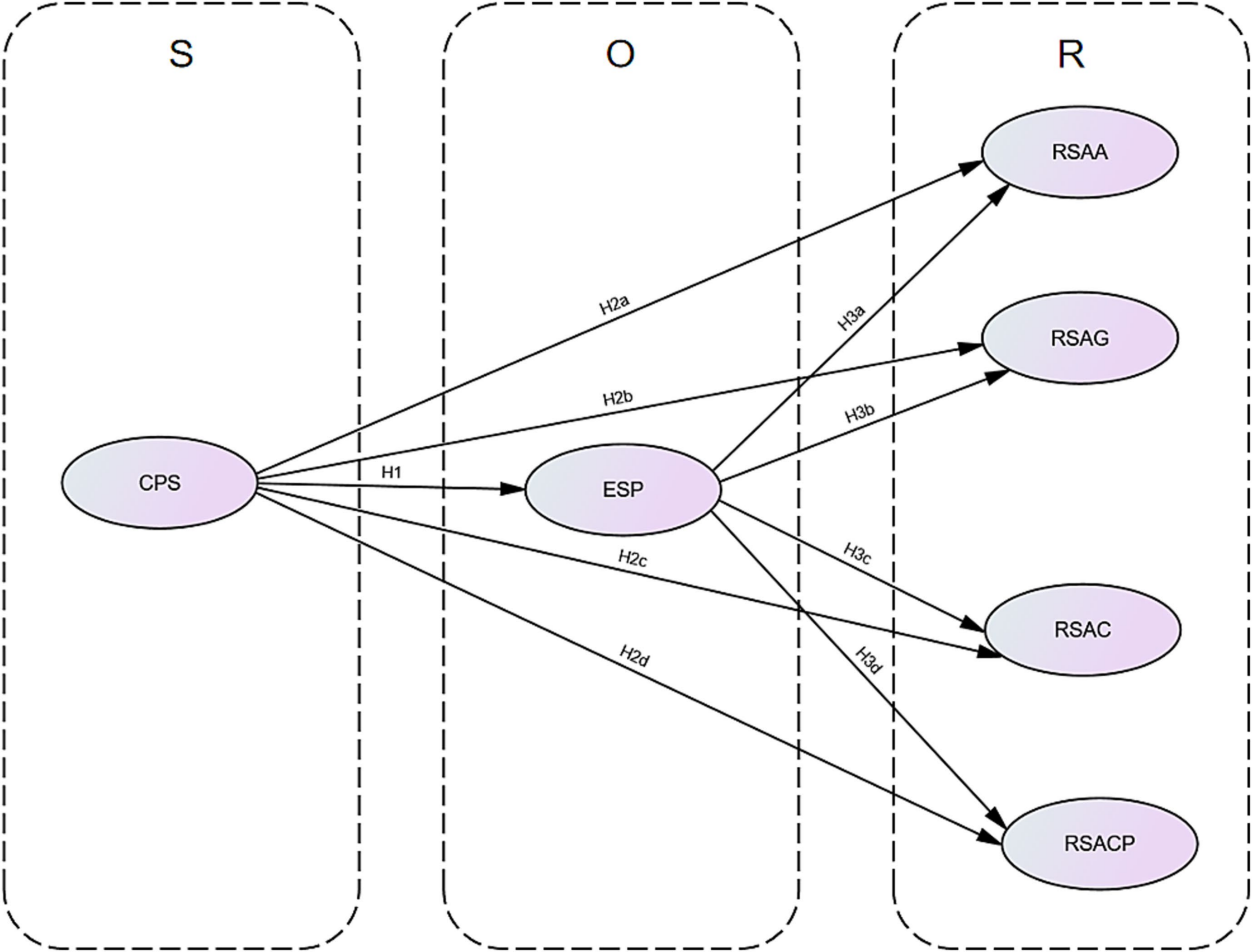
Research model. CPS = urban park soundscape cognitive image; ESP = emotional pleasure; RSAA = being away; RSAG = fascination; RSAC = coherence; RSACP = compatibility.

According to the SOR framework, an environmental stimulus may be associated with a response through an organismic psychological state (35). In the present study, soundscape cognitive image represents visitors’ cognitive evaluation of the park sound environment, emotional pleasure represents their internal affective state, and perceived restorativeness represents the restorative response. The above literature indicates that soundscape cognition may be related to emotional pleasure (41–43, 50), while emotional pleasure and place-related affective experiences may be related to perceived restorativeness (49, 52) (Figure 1). Therefore, emotional pleasure may constitute a potential psychological pathway linking soundscape cognitive image and perceived restorativeness. Accordingly, the following hypotheses are proposed:

*H4a*: Emotional pleasure mediates the relationship between soundscape cognitive image and being away.

*H4b*: Emotional pleasure mediates the relationship between soundscape cognitive image and fascination.

*H4c*: Emotional pleasure mediates the relationship between soundscape cognitive image and coherence.

*H4d*: Emotional pleasure mediates the relationship between soundscape cognitive image and compatibility.

## 3 Materials and methods

### 3.1 Study area and data collection

The questionnaire consisted of two parts. The first part explained the purpose of the study, stated that respondents’ data would be kept strictly confidential, and emphasized that the collected information would be used only for scientific research and not for any commercial purposes. The second part included measurement items for the study variables. The order of the items was randomly arranged to reduce respondent bias.

This study selected 12 urban parks in Foshan, China, as survey sites. The sites covered comprehensive parks, cultural and community parks, waterfront parks, mountain ecological parks, and large landscape parks. Respondents were recruited using an on-site intercept sampling approach with purposive coverage of different park types and functional zones. Surveyors approached visitors in quiet resting areas, public activity areas, waterside or natural landscape areas, and walking or fitness areas. A total of 313 questionnaires were distributed, and 300 valid responses were retained after excluding incomplete or logically inconsistent responses, yielding an effective response rate of 95.8%. Because the exact number of valid questionnaires collected in each park and on each survey day was not fully retained for all sites, park-level sample quotas and response distributions cannot be reported. The analysis therefore uses the 300 valid questionnaires as a pooled visitor-level sample, and this limitation is explicitly acknowledged in Section 5.4.

Detailed characteristics of the surveyed parks are provided in Supplementary Table S1. The field survey was conducted during the fieldwork period from May to August 2025.

### 3.2 Questionnaire design and measures

The measurement section of the questionnaire included items for urban park soundscape cognitive image, emotional pleasure, and perceived restorativeness, together with basic sociodemographic and recreational-use information. All construct items were adapted from established scales and revised to fit the context of urban park soundscape experience. A five-point Likert scale was used for the core constructs, ranging from 1 = strongly disagree to 5 = strongly agree. Before the formal field survey, a pilot test with 30 respondents was conducted to examine item clarity and scale reliability. Ambiguous wording was revised according to the pilot feedback.

### 3.3 Measurement of soundscape cognitive image

Soundscape cognitive image was used to capture visitors’ integrated subjective evaluation of the urban park sound environment. Drawing on the cognitive image scale of Zhang et al. (57), this study adapted four items to the soundscape context of urban parks. These items assessed visitors’ perceptions of acoustic comfort, pleasantness, harmony with the park landscape, and suitability for leisure expectations. This construct therefore represents a perception-based appraisal of the park soundscape rather than an objective acoustic measurement.

### 3.4 Measurement of emotional pleasure

This study was theoretically grounded in the pleasure dimension of the Mehrabian and Russell environmental emotion framework (58). During the preliminary stage, both emotional pleasure and emotional arousal were considered as possible emotional-state dimensions. However, subsequent data screening and model-fitting checks indicated that emotional arousal did not show sufficient compatibility with the formal model. Therefore, the formal analysis included emotional pleasure as the emotional-state variable. Emotional pleasure was measured using four semantic-differential items adapted to the urban park soundscape context.

### 3.5 Measurement of perceived restorativeness

This study was based on Attention Restoration Theory, which states that environments with four core characteristics, namely being away, fascination, coherence, and compatibility, have psychological restorative functions. Referring to the environmental restorativeness scale revised by Zhang and Liu (59) in the context of urban recreation in China, this study measured perceived restorativeness from four dimensions: being away, fascination, coherence, and compatibility. The retained measurement items and sources are reported in Supplementary Table S2.

### 3.6 Data analysis

Python 3.10 was used for data coding, cleaning, and descriptive statistical analysis. Reliability and convergent validity were evaluated using standardized factor loadings, Cronbach’s α, composite reliability (CR), average variance extracted (AVE), and KMO values. Discriminant validity was assessed using the Fornell–Larcker criterion. Common method bias was examined using an unrotated principal component analysis. Confirmatory factor analysis, structural equation modeling, and mediation tests were conducted using AMOS 26.0. The mediating role of emotional pleasure was tested using a bias-corrected Bootstrap procedure with 5,000 resamples and 95% confidence intervals. Because the data were cross-sectional and self-reported, path coefficients and mediation results were interpreted as statistical associations rather than causal evidence.

## 4 Results

### 4.1 Sample characteristics

As shown in Table 1, the gender distribution was relatively balanced, with males accounting for 54.00% and females accounting for 46.00%. Respondents were mainly aged 18–35 years, indicating that younger visitors constituted the largest age group in the sample. The corrected demographic statistics were calculated using the 300 valid questionnaires as the denominator.

**Table 1:** Demographic characteristics of the sample.

Category		Number	Percentage
Gender	Male	162	54.00%
	Female	138	46.00%
Age	18–35	188	62.67%
	36–45	31	10.33%
	46–55 years	34	11.33%
	56 and over	47	15.67%
Mode of transport	On foot	114	38.00%
	Cycling	42	14.00%
	Electric vehicles	55	18.33%
	Public transport	56	18.67%
	Driving	33	11.00%
Occupation	Civil servant	4	1.30%
	Corporate and institutional managers	8	2.70%
	Professionals and technical staff in education and culture	17	5.70%
	Service, sales, and trade personnel	25	8.30%
	Workers	13	4.30%
	Farmers	3	1.00%
	Military	0	0.00%
	Students	119	39.70%
	Retired staff	46	15.33%
	Other occupations	65	21.70%
Educational attainment	Primary school or below	17	5.70%
	Lower secondary	64	21.30%
	Upper secondary/vocational school	90	30%
	College/undergraduate	127	42.30%
	Postgraduate	2	0.70%
Monthly income	≤5,000 yuan	199	66.33%
	5,001–10,000 yuan	46	15.33%
	10,001–15,000 yuan	40	13.33%
	15,001–20,000 yuan	10	3.33%
	20,001–25,000 yuan	2	0.67%
	>25,000 yuan	3	1.00%

Regarding educational attainment, respondents with a college or undergraduate degree accounted for the largest proportion (42.30%), followed by those with a senior high school or secondary vocational education (30.00%). This suggests that the educational level of the respondents was mainly concentrated at the middle and higher levels. Students accounted for the largest occupational group (39.70%), followed by retirees (15.33%), suggesting that visitors with relatively flexible leisure time formed an important part of the sample.

Monthly income was mainly 5,000 yuan or below (66.33%), followed by 5,001–10,000 yuan (15.33%). This pattern is consistent with the relatively high proportion of students in the sample. Walking was the most common mode of transportation to the parks (38.00%), followed by public transportation (18.67%) and electric bicycles (18.33%), suggesting that many respondents were nearby residents or visitors making short-distance trips.

### 4.2 Reliability and convergent validity

As shown in Table 2, the standardized factor loadings of the items in the formal measurement model ranged from 0.610 to 0.934, exceeding the recommended threshold of 0.500. The composite reliability values of all constructs ranged from 0.815 to 0.899, and the average variance extracted values ranged from 0.515 to 0.695. The Cronbach’s alpha coefficients for soundscape cognitive image, emotional pleasure, and perceived restorativeness were all above 0.800. The KMO values for the soundscape cognitive image, emotional pleasure, and perceived restorativeness scales were 0.777, 0.812, and 0.871, respectively. These results indicate that the measurement model had acceptable internal consistency, construct reliability, and convergent validity.

**Table 2:** Reliability and validity of the measurement model.

Latent variable	Observed variable	Factor loading	CR	AVE	Cronbach’s α	KMO	Total variance explained
Urban park soundscape cognitive image (CPS)	CPS1	0.876	0.857	0.600	0.853	0.777	69.647%
	CPS2	0.843					
	CPS3	0.835					
	CPS4	0.781					
Emotional pleasure (ESP)	ESP1	0.877	0.899	0.695	0.889	0.812	76.038%
	ESP2	0.917					
	ESP3	0.934					
	ESP4	0.748					
Being away (RSAA)	RSAA1	0.673	0.840	0.515	0.868	0.871	65.634%
	RSAA2	0.755					
	RSAA3	0.759					
	RSAA4	0.774					
	RSAA5	0.649					
Fascination (RSAG)	RSAG1	0.610	0.842	0.572			
	RSAG2	0.736					
	RSAG3	0.797					
	RSAG4	0.828					
Coherence (RSAC)	RSAC2	0.875	0.815	0.530			
	RSAC3	0.768					
	RSAC4	0.834					
	RSAC6	0.688					
Compatibility (RSACP)	RSACP2	0.788	0.838	0.548			
	RSACP3	0.768					
	RSACP4	0.755					
	RSACP5	0.757					

CPS = urban park soundscape cognitive image; ESP = emotional pleasure; RSAA = being away; RSAG = fascination; RSAC = coherence; RSACP = compatibility.

### 4.3 Discriminant validity

As shown in Table 3, discriminant validity was assessed by comparing the square root of the average variance extracted (AVE) for each construct with the correlations between that construct and the other constructs. The diagonal values, representing the square roots of AVE, ranged from 0.718 to 0.834. For each construct, the square root of AVE was greater than all corresponding interconstruct correlations in the same row and column, indicating acceptable discriminant validity among CPS, ESP, RSAA, RSAG, RSAC, and RSACP. For example, the square root of AVE for CPS was 0.775, which exceeded its correlations with ESP (0.287), RSAA (0.646), RSAG (0.509), RSAC (−0.038), and RSACP (0.642). Similarly, the square root of AVE for RSAA was 0.718 and exceeded its correlations with RSAG (0.667), RSACP (0.632), and the other constructs. These results indicate that the latent constructs measured distinct aspects of soundscape cognitive image, emotional pleasure, and perceived restorativeness.

**Table 3:** Discriminant validity.

Variable	CPS	ESP	RSAA	RSAG	RSAC	RSACP
CPS	0.775					
ESP	0.287	0.834				
RSAA	0.646	0.364	0.718			
RSAG	0.509	0.231	0.667	0.756		
RSAC	−0.038	−0.247	0.100	0.159	0.728	
RSACP	0.642	0.345	0.632	0.611	0.112	0.740

Diagonal values are the square roots of AVE. CPS = urban park soundscape cognitive image; ESP = emotional pleasure; RSAA = being away; RSAG = fascination; RSAC = coherence; RSACP = compatibility.

### 4.4 Common method bias

All measurement items in the formal research model were included in an unrotated principal component analysis. Six factors with eigenvalues greater than 1 were extracted, and the cumulative variance explained was 68.432%. The first factor explained 31.796% of the variance, which was below the 50% threshold. Therefore, no serious common method bias was detected in the dataset (Table 4).

**Table 4:** Common method bias test.

Component	Initial eigenvalue: total	Initial eigenvalue: % variance	Initial eigenvalue: cumulative %	Extraction SS loadings: total	Extraction SS loadings: % variance	Extraction SS loadings: cumulative %
1	7.949	31.796	31.796	7.949	31.796	31.796
2	3.282	13.126	44.922	3.282	13.126	44.922
3	1.920	7.678	52.600	1.920	7.678	52.600
4	1.491	5.965	58.566	1.491	5.965	58.566
5	1.329	5.318	63.884	1.329	5.318	63.884
6	1.137	4.549	68.432	1.137	4.549	68.432
7	0.790	3.158	71.591			
8	0.700	2.801	74.392			
9	0.640	2.560	76.952			

### 4.5 Model fit

The measurement model and mediation model showed acceptable fit. For the confirmatory factor analysis model, χ^2^/df = 1.649, GFI = 0.899, AGFI = 0.874, CFI = 0.956, TLI = 0.949, and RMSEA = 0.047. For the mediation model, χ^2^/df = 1.951, GFI = 0.876, AGFI = 0.848, CFI = 0.934, TLI = 0.925, and RMSEA = 0.056. These indices indicate that both the measurement model and the mediation structural model achieved acceptable fit and were adequate for subsequent path and mediation analyses (Table 5).

**Table 5:** Model fit indices.

Index	Criterion	CFA measurement model	Mediation structural model
χ^2^ (CMIN)	Lower values indicate better fit	428.823	519.032
df	Reported for model parsimony	260	266
χ^2^/df	<3 acceptable to good; 3–5 acceptable	1.649	1.951
GFI	>0.80 acceptable; >0.90 good	0.899	0.876
AGFI	>0.80 acceptable; >0.90 good	0.874	0.848
CFI	>0.80 acceptable; >0.90 good	0.956	0.934
TLI (NNFI)	>0.80 acceptable; >0.90 good	0.949	0.925
RMSEA	<0.08 good; <0.10 acceptable	0.047	0.056

Commonly used SEM fit criteria were adopted. Because several indices, especially GFI and AGFI, were below the stricter 0.90 criterion, the model fit was described as acceptable rather than excellent.

The full CFA diagram is provided in Supplementary Figure S1.

### 4.6 Direct path analysis

The direct path results are shown in Table 6. Soundscape cognitive image was positively associated with emotional pleasure (β = 0.297, *p* < 0.001), supporting H1. Soundscape cognitive image was also positively associated with being away (β = 0.659, *p* < 0.001), fascination (β = 0.578, *p* < 0.001), and compatibility (β = 0.658, *p* < 0.001), supporting H2a, H2b, and H2d. However, its relationship with coherence was not significant (β = 0.103, *p* = 0.133), so H2c was not supported. Emotional pleasure was positively associated with being away (β = 0.170, *p* < 0.01) and compatibility (β = 0.151, *p* < 0.01), supporting H3a and H3d. Its relationship with fascination was not significant (β = 0.062, *p* = 0.296), so H3b was not supported. Emotional pleasure was significantly but negatively associated with coherence (β = −0.273, *p* < 0.001), which was inconsistent with the hypothesized positive direction; therefore, H3c was not supported in its original direction.

**Table 6:** Direct effect path analysis results.

Path	Unstd.	S. E.	C. R.	*p*	Std. β	*R* ^2^
ESP ← CPS	0.256	0.059	4.352	<0.001	0.297	0.088
RSAA ← CPS	0.641	0.076	8.464	<0.001	0.659	0.530
RSAA ← ESP	0.192	0.063	3.045	<0.01	0.170	
RSAG ← CPS	0.555	0.076	7.288	<0.001	0.578	0.359
RSAG ← ESP	0.069	0.066	1.045	0.296	0.062	
RSAC ← CPS	0.122	0.081	1.504	0.133	0.103	0.069
RSAC ← ESP	−0.374	0.096	−3.917	<0.001	−0.273	
RSACP ← CPS	0.711	0.082	8.668	<0.001	0.658	0.516
RSACP ← ESP	0.189	0.070	2.694	<0.01	0.151	

CPS = urban park soundscape cognitive image; ESP = emotional pleasure; RSAA = being away; RSAG = fascination; RSAC = coherence; RSACP = compatibility. Standard significance notation.

The structural model diagram is provided in Supplementary Figure S2.

### 4.7 Mediation analysis of emotional pleasure

A bias-corrected Bootstrap procedure with 5,000 resamples was used to test the mediating role of emotional pleasure. The indirect pathway from soundscape cognitive image to being away through emotional pleasure was significant (indirect effect = 0.049, 95% CI [0.007, 0.097]), supporting H4a. The indirect pathway for fascination was not significant (indirect effect = 0.018, 95% CI [−0.017, 0.058]); therefore, H4b was not supported. The indirect pathway for coherence was significant but negative (indirect effect = −0.096, 95% CI [−0.175, −0.042]). Because this direction was inconsistent with the hypothesized positive mediation and the total relationship was not significant, H4c was not supported as originally hypothesized. This result is reported as an unexpected negative indirect association and should be interpreted cautiously. The indirect pathway for compatibility was significant (indirect effect = 0.048, 95% CI [0.004, 0.104]), supporting H4d. Overall, emotional pleasure mainly connected soundscape cognitive image with being away and compatibility, whereas its role in fascination was not significant and its relationship with coherence was negative and dimension-specific (Table 7).

**Table 7:** Test of the mediating effect of emotional pleasure.

Path	Effect size	Lower Boot CI	Upper Boot CI	*p*	Result
CPS → ESP → RSAA	0.049	0.007	0.097		Supported
CPS → RSAA	0.641	0.474	0.842	<0.001	Supported
Total 1	0.690	0.515	0.892	<0.001	Supported
CPS → ESP → RSAG	0.018	−0.017	0.058		Not supported
CPS → RSAG	0.555	0.403	0.732	<0.001	Supported
Total 2	0.573	0.425	0.746	<0.001	Supported
CPS → ESP → RSAC	−0.096	−0.175	−0.042		Unexpected negative indirect association; H4c not supported as hypothesized
CPS → RSAC	0.122	−0.070	0.313	0.133	Not supported
Total 3	0.026	−0.157	0.203	>0.05	Not supported
CPS → ESP → RSACP	0.048	0.004	0.104		Supported
CPS → RSACP	0.711	0.555	0.896	<0.001	Supported
Total 4	0.759	0.603	0.951	<0.001	Supported

Blank p cells for indirect effects were evaluated according to whether the 95% Bootstrap confidence interval included zero. The coherence indirect pathway was significant but negative and should be interpreted cautiously.

## 5 Discussion

### 5.1 Key findings

These findings are generally consistent with the restorative soundscape literature reviewed above, but they also extend it in two aspects. First, rather than examining sound preference or audio-visual interaction alone, this study focuses on visitors’ overall cognitive image of urban park soundscapes, including comfort, pleasantness, harmony, and contextual suitability. Second, by distinguishing being away, fascination, coherence, and compatibility, the findings show that soundscape-related restoration is dimension-specific rather than uniform. In particular, emotional pleasure was more clearly linked to being away and compatibility than to fascination, while its negative association with coherence suggests that pleasant but heterogeneous sound sources in multi-functional parks may not always strengthen perceived order or consistency. Therefore, the contribution of this study lies not simply in confirming that pleasant sounds are preferred, but in clarifying how soundscape cognition and emotional pleasure are selectively associated with different restorative perceptions.

This study examined how recreational visitors’ cognitive image of urban park soundscapes is associated with perceived restorativeness through emotional pleasure. Based on survey data from 300 visitors in 12 urban parks in Foshan, the SEM results show that soundscape cognitive image is positively associated with emotional pleasure. It is also positively associated with being away, fascination, and compatibility, while its direct relationship with coherence is not significant. Emotional pleasure is positively associated with being away and compatibility, is not significantly associated with fascination, and is negatively associated with coherence. These results suggest that favorable soundscape cognition is not uniformly related to all restorative dimensions. Instead, the strongest and most consistent relationships appear in visitors’ sense of psychological escape and person–environment fit. The negative path from emotional pleasure to coherence may reflect the coexistence of pleasant but heterogeneous activity sounds in multi-functional parks, but this interpretation should remain cautious because the study is cross-sectional and based on subjective reports.

Before interpreting the unexpected negative association between emotional pleasure and coherence, the retained coherence items were rechecked for item direction, translation, and reverse coding. No reverse-coded item was retained in the formal coherence measurement, and the item direction was consistent with the intended construct meaning. Therefore, the negative path was reported as an empirical result rather than treated as support for the originally hypothesized positive relationship. One possible explanation is that in multi-functional urban parks, pleasant experiences may be produced by lively and heterogeneous sound sources, such as social interaction, children’s play, flowing water, birdsong, or occasional music. Such sounds may enhance pleasure but may also reduce the perceived order, clarity, or consistency of the overall acoustic environment. This interpretation remains tentative and should be verified in future studies using objective acoustic indicators and field observations.

### 5.2 Theoretical contributions

First, this study applies the SOR framework to restorative soundscape research by linking soundscape cognitive image, emotional pleasure, and perceived restorativeness in an integrated model. This helps move beyond a simple direct-association approach and highlights the role of visitors’ internal affective state in soundscape-related restoration. Second, this study clarifies that emotional pleasure is a selective rather than uniform pathway. Emotional pleasure was related to being away and compatibility, but it was not related to fascination and showed a negative relationship with coherence. This finding suggests that restorative soundscape evaluation should distinguish among different dimensions of perceived restorativeness. Third, by defining soundscape cognitive image as a subjective appraisal of comfort, pleasantness, harmony, and contextual suitability, this study connects destination-image-based measurement logic with perception-based soundscape theory, while also acknowledging that subjective soundscape cognition cannot replace objective acoustic indicators.

### 5.3 Practical implications

The findings provide several cautious implications for urban park soundscape evaluation and management.

First, urban park soundscape management may consider emotional pleasure as one important evaluation dimension, rather than treating it as the sole design goal. Enhancing visitors’ emotional pleasure can be considered alongside perceived comfort, harmony, contextual suitability, and objective acoustic conditions. To support pleasant soundscape experiences, existing natural soundscape resources should first be identified and protected. For example, core ecological areas may be designated as soundscape conservation zones, and natural water systems, native vegetation, and wildlife habitats may be maintained to support natural sound sources such as birdsong, flowing water, and wind. Second, negative noise interference can be managed through context-sensitive strategies rather than uniform control targets. Plant buffer belts composed of trees, shrubs, and grasses may help reduce surrounding traffic noise, and quiet resting areas should be evaluated through both visitor perception and objective acoustic monitoring. Third, audiovisual experience can be considered in selected landscape nodes. In landscape-node design, soundscapes and visual landscapes may be evaluated together to improve the overall visitor experience. For example, aquatic plants and waterside platforms may be arranged in water landscape areas to support visitors’ experience of waterside soundscapes, but such design decisions should be further verified by acoustic measurement and field observation.

Second, differentiated soundscape zoning may be considered according to the restorative goals of different functional areas. Soundscape strategies can be adjusted for quiet resting areas, waterside or natural areas, public activity areas, and walking or fitness routes. In stress-relief areas, such as meditation spaces and quiet walking trails, managers may prioritize low-disturbance sound environments that support visitors’ sense of being away. Commercial broadcasts and amusement-facility noise may be limited in these areas to maintain an environment dominated by natural sounds. In nature-experience areas, such as science education gardens and wetland landscapes, soundscape interpretation boards or natural-sound observation facilities may be introduced as exploratory tools to enrich visitors’ soundscape awareness. In public activity areas, such as squares and fitness spaces, managers may focus on the compatibility between soundscape conditions and activity needs. Spatial separation and time-based management may be combined to reduce interference between active and quiet zones.

### 5.4 Limitations and future research

Despite its theoretical and practical contributions, this study still has several limitations. First, this study adopted a cross-sectional self-report questionnaire design, which can reveal statistical associations and potential mediating pathways among variables but cannot establish strict causal relationships. Future studies could use longitudinal tracking designs, field experiments, or controlled audiovisual simulations to further examine the directionality of the observed associations and the potential pathways linking soundscape cognitive image to perceived restorativeness.

Second, although the survey covered 12 parks and several functional zones, the exact number of valid questionnaires collected in each park and on each survey day was not fully retained. Therefore, park-level representativeness and nested park-level effects could not be evaluated. Future research should record park-level sample sizes, survey dates, time periods, and functional zones more systematically and should adopt cluster-robust or multilevel modeling when the data structure permits.

Third, the measurement of soundscapes in this study focused mainly on overall subjective evaluation. It did not distinguish the heterogeneous effects of natural sounds, human sounds, and mechanical sounds, nor did it incorporate objective physical measurements using sound level meters or other acoustic instruments. Future studies could refine the measurement of soundscape types and acoustic attributes by combining subjective perception data with objective acoustic indicators.

Fourth, the sample was concentrated in Foshan in the Pearl River Delta region, and students accounted for a relatively high proportion of the respondents. Therefore, the generalizability of the findings needs to be further tested across different regions and population groups. Future research could expand the sample to cities with different climatic, cultural, and spatial characteristics and compare the restorative effects of urban park soundscapes among different age groups, occupations, and cultural backgrounds.

Finally, this study did not examine the interaction between soundscapes and other sensory stimuli, such as visual and olfactory experiences. Urban park restoration is often shaped by multisensory environmental perception. Future studies could further explore the interactive effects of sound, vision, smell, and other sensory dimensions, and extend the research to multisensory integration and cross cultural comparison. These efforts would help improve the theoretical system and practical application of soundscape health benefits.

## 6 Conclusion

This study examined the associations among urban park soundscape cognitive image, emotional pleasure, and recreational visitors’ perceived restorativeness. Based on survey data from 300 visitors in 12 urban parks in Foshan, the SEM results showed that soundscape cognitive image was positively associated with emotional pleasure and with the being away, fascination, and compatibility dimensions of perceived restorativeness. Its direct relationship with coherence was not significant. Emotional pleasure was positively associated with being away and compatibility, was not significantly associated with fascination, and was negatively associated with coherence. Bootstrap results further indicated that emotional pleasure constituted a significant indirect pathway between soundscape cognitive image and being away and compatibility. A significant but negative indirect pathway was found for coherence, while no significant indirect pathway was found for fascination. These findings suggest that the relationship between soundscape cognition, emotional pleasure, and perceived restorativeness is dimension-specific rather than uniform. Overall, this study extends the SOR framework to urban park soundscape research and provides association-based evidence for health-oriented soundscape evaluation and planning. Because the data were cross-sectional and self-reported, the conclusions should be interpreted cautiously and verified through future longitudinal, experimental, and objective acoustic studies.

## Data Availability

The original contributions presented in the study are included in the article/Supplementary material, further inquiries can be directed to the corresponding authors.
