# Supplementary material for: Linking urban park soundscape cognitive image to recreational visitors’ perceived restorativeness: the mediating role of emotional pleasure
Source: Front Public Health. 2026 Jul 15;14:1891815. doi: 10.3389/fpubh.2026.1891815 (PMC13415582; doi:10.3389/fpubh.2026.1891815)

## Figure S1. Confirmatory Factor Analysis Measurement Model


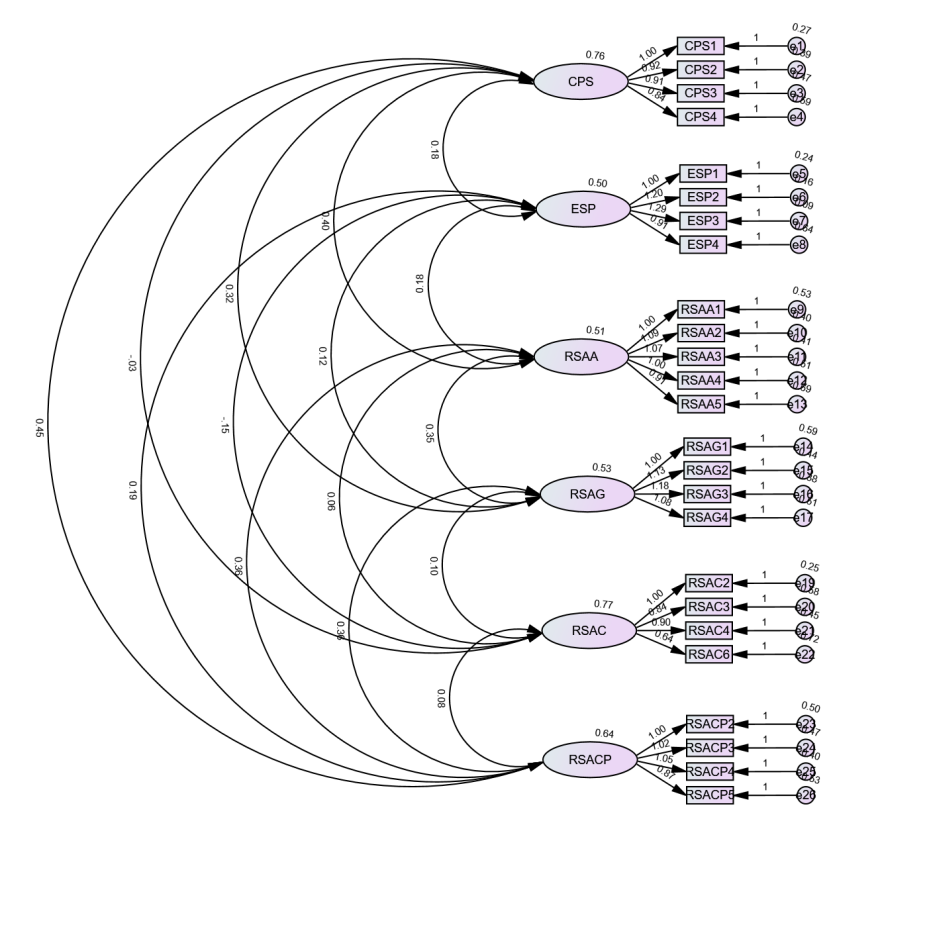


.

## Figure S2. Mediation Structural Model


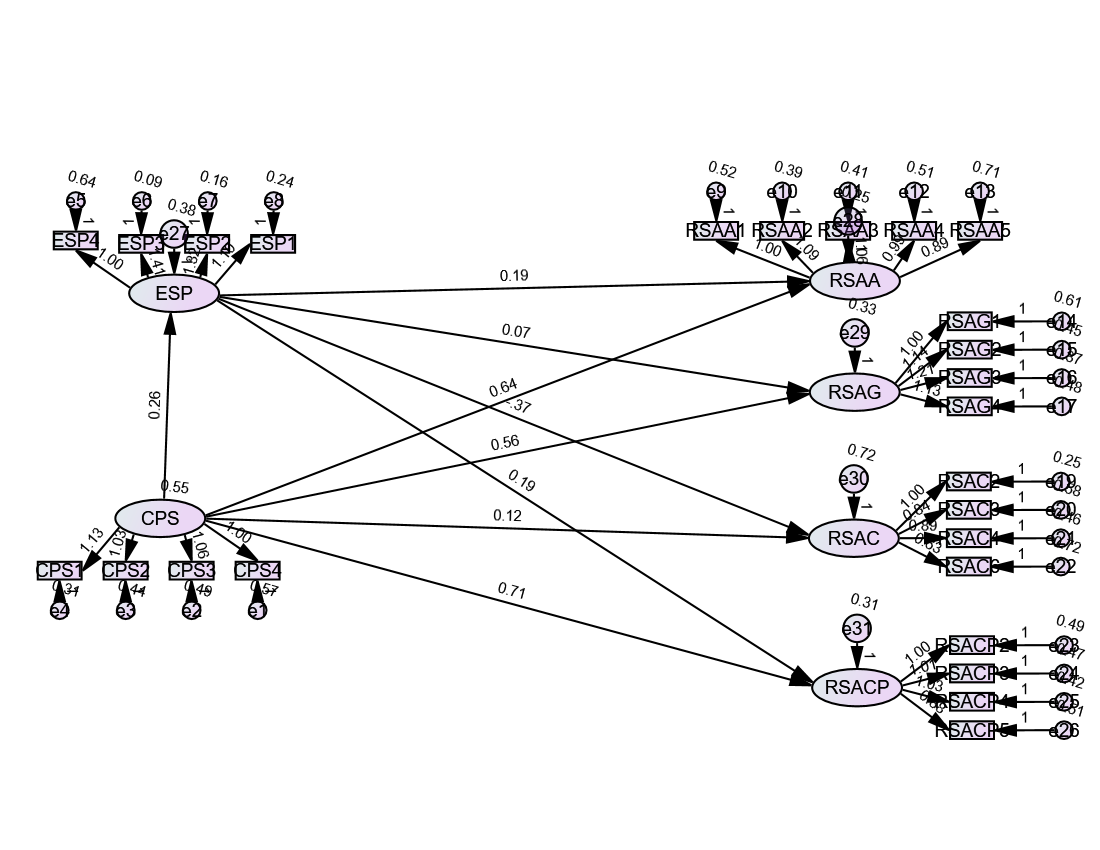

Supplement: Supplementary file 1 [file Data_Sheet_1.docx]
