# Supplementary material for: Linking urban park soundscape cognitive image to recreational visitors’ perceived restorativeness: the mediating role of emotional pleasure
Source: Front Public Health. 2026 Jul 15;14:1891815. doi: 10.3389/fpubh.2026.1891815 (PMC13415582; doi:10.3389/fpubh.2026.1891815)
Supplement: Supplementary file 2 [file Table_1.docx]

Supplementary Table S1. Characteristics of the Surveyed Urban Parks

| **Surveyed park** | **Location** | **Core feature** | **Area (ha)** | **Functional zones** | **Soundscape characteristics** |
| --- | --- | --- | --- | --- | --- |
| Wenhua Park | Chancheng District | Lingnan garden; water-dance light show | Approx. 61 | Lawns, waterfront areas, children’s areas, sports areas, flower fields, activity areas | Natural sounds as the base; performance music on holidays; dynamic and quiet zones are separated |
| Asia Arts Park | Chancheng District | Lingnan water-town and arts theme; Lotus Island as a landmark | Approx. 40 | Squares, water forest, Lotus Island, dragon-boat area | Flowing water and birdsong predominate; quiet during mist-spray periods; light music during morning exercises |
| Shiwan Park | Chancheng District | Ceramic culture theme with a lakeside layout | Approx. 16.3 | Ceramic exhibition, mountain-water landscapes, cultural-creative areas, amusement areas, walking paths | Birdsong in woodland and lake-water sounds; explanations during cultural activities |
| Zhongshan Park | Chancheng District | Long-established comprehensive park in Foshan | Approx. 28.07 | Waterscapes, historical areas, zoo, amusement areas, fitness areas | Amusement areas are noisy; quiet areas have richer natural sounds; strong dynamic–quiet contrast |
| Yunshan Park | Danzao Town, Nanhai District | Mountain ecological park and urban green lung | Approx. 150 | Squares, hiking trails, lotus pond, sports areas | Mountain-forest natural sounds predominate; occasional voices on trails; overall quiet atmosphere |
| Zhanqifeng Ecological Park | Lishui Town, Nanhai District | Mountain ecological recreation park with Zhanqifeng as a landmark | Approx. 215.76 | Hiking trails, hilltop square, temple, leisure areas | Quiet mountain-forest soundscape; occasional temple bells; tranquil atmosphere |
| Huangqi Park | Dali Town, Nanhai District | Waterfront leisure and Longmu culture; linear riverside layout | No precise statistics available | Waterfront sports, leisure recreation, cultural areas, greenways | Riverside wind and water sounds; folk drums and gongs during festivals; strong everyday-life atmosphere |
| Jiulong Park | Dali Town, Nanhai District | All-age parent–child park with amusement facilities | Approx. 8 | Amusement areas, landscape lake, lawns, fitness areas | Lively and noisy amusement areas; quiet lake area; clear dynamic–quiet separation |
| Lunjiao Binjiang Park | Lunjiao Subdistrict, Shunde District | Riverside ecological leisure belt and key section of Baili Fanghua | Core section approx. 12 | Running tracks, viewing corridors and bridges, waterside boardwalks, lawns | River wind and water sounds as the base; occasional boat horns; open and relaxed atmosphere |
| Shunfengshan Park | Daliang Subdistrict, Shunde District | Iconic mountain-water comprehensive park in Foshan | Approx. 547.3 | Paifang square, lake area, ancient tower, gardens, wetlands | Natural sounds dominate mountain and lake areas; activity sounds in squares; rich soundscape layers |
| Huaxi Park | Ronggui Subdistrict, Shunde District | Ecological leisure comprehensive park | Approx. 13.8 | Squares, art gallery, flower borders, fitness areas | Birdsong and wind in shaded areas; fitness sounds in sports areas; everyday-life atmosphere |
| Wenta Park | Ronggui Subdistrict, Shunde District | Cultural leisure park centered on a Qing-dynasty literary pagoda | No precise statistics available | Wenta cultural area, sports areas, walking paths, squares | Quiet around the pagoda; occasional instrumental music; active atmosphere in sports areas |

Supplementary Table S2. Measurement items and sources

| **Item** | **Measurement item** | **Source** |
| --- | --- | --- |
| CPS1 | The sound environment of this park makes me feel comfortable. | Zhang et al. [57], adapted |
| CPS2 | The sounds in this park are pleasant. |  |
| CPS3 | The sound environment is harmonious with the park landscape. |  |
| CPS4 | Overall, the soundscape of this park meets my leisure expectations. |  |
| ESP1 | When perceiving the soundscape, I feel pleasant. | Mehrabian & Russell [58], adapted |
| ESP2 | When perceiving the soundscape, I feel relaxed. |  |
| ESP3 | When perceiving the soundscape, I feel comfortable. |  |
| ESP4 | When perceiving the soundscape, I feel satisfied. |  |
| RSAA1 | The park environment makes me feel away from my daily routine. | Zhang & Liu [59], adapted |
| RSAA2 | The park environment allows me to temporarily escape from everyday work or study. |  |
| RSAA3 | The park environment helps me forget worries or pressure. |  |
| RSAA4 | The park environment makes me feel away from stressful situations. |  |
| RSAA5 | The park environment gives me a sense of being in a different place. |  |
| RSAG1 | The park environment attracts my attention effortlessly. |  |
| RSAG2 | The park environment is interesting to me. |  |
| RSAG3 | The park environment arouses my curiosity to explore. |  |
| RSAG4 | The park environment is attractive to me. | Zhang & Liu [56], adapted |
| RSAC2 | The park environment feels orderly. | Zhang & Liu [59], adapted |
| RSAC3 | The park environment has a clear structure. |  |
| RSAC4 | The different environmental elements in the park fit together consistently. |  |
| RSAC6 | The park environment is easy to understand. |  |
| RSACP2 | The park environment fits my recreational needs. |  |
| RSACP3 | The park environment fits my activity goals in the park. |  |
| RSACP4 | The park environment matches my current mood. |  |
| RSACP5 | The park environment fits my preferred way of using the park. |  |
